# Aortic ^18^F-FDG uptake in patients suffering from granulomatosis with polyangiitis

**DOI:** 10.1007/s00259-015-3081-y

**Published:** 2015-05-21

**Authors:** Michael J. Kemna, Jan Bucerius, Marjolein Drent, Stefan Vöö, Martine Veenman, Pieter van Paassen, Jan Willem Cohen Tervaert, Marinus J. P. G. van Kroonenburgh

**Affiliations:** Department of Nephrology and Clinical Immunology, Maastricht University Medical Center, Maastricht, The Netherlands; Cardiovascular Research Institute Maastricht (CARIM), Maastricht University, Maastricht, The Netherlands; Department of Nuclear Medicine, Maastricht University Medical Center, Maastricht, The Netherlands; Department of Nuclear Medicine, University Hospital RWTH Aachen, Aachen, Germany; Department of Pharmacology and Toxicology, Maastricht University, Maastricht, The Netherlands; Noordoever Academy, Sint Franciscus Gasthuis, Rotterdam, The Netherlands

**Keywords:** Positron emission tomography scanning, PET/CT, Aortic inflammation, Granulomatosis with polyangiitis, ANCA-associated vasculitis, Sarcoidosis, Large vessel vasculitis, Giant cell arteritis

## Abstract

**Purpose:**

The objective of the study was to systematically assess aortic inflammation in patients with granulomatosis with polyangiitis (GPA) using ^18^F-2-deoxy-2-[^18^F]fluoro-D-glucose (FDG) positron emission tomography (PET)/CT.

**Methods:**

Aortic inflammation was studied in PET/CT scans obtained from 21 patients with GPA; 14 patients with sarcoidosis were included as disease controls, 7 patients with stage I or II head and neck carcinoma ascertained during routine clinical practice were used as healthy controls (HC) and 5 patients with large vessel vasculitis (LVV) were used as positive controls. Aortic ^18^F-FDG uptake was expressed as the blood-normalized maximum standardized uptake value (SUV_max_), known as the target to background ratio (mean TBR_max_).

**Results:**

The mean TBR_max_ (interquartile range) of the aorta in patients with GPA, sarcoidosis, HC and LVV were 1.75 (1.32–2.05), 1.62 (1.54–1.74), 1.29 (1.22–1.52) and 2.03 (1.67–2.45), respectively. The mean TBR_max_ was significantly higher in patients suffering from GPA or LVV compared to HC (*p* < 0.05 and *p* < 0.005, respectively) and tended to be higher in patients suffering from sarcoidosis, but this did not reach statistical significance (*p* = 0.098). The mean TBR_max_ of the most diseased segment was significantly higher compared to HC [1.57 (1.39–1.81)] in LVV patients [2.55 (2.22–2.82), *p* < 0.005], GPA patients [2.17 (1.89–2.83), *p* < 0.005] and patients suffering from sarcoidosis [2.04 (1.88–2.20), *p* < 0.05]. In GPA patients, the mean TBR_max_ of the aorta was significantly higher in patients with previous renal involvement [2.01 (1.69–2.53)] compared to patients without renal involvement in the past [1.60 (1.51–1.80), *p* < 0.05]. Interrater reproducibility with a second reader was high (all intraclass correlation coefficients >0.9).

**Conclusion:**

Patients suffering from GPA show marked aortic FDG uptake.

**Electronic supplementary material:**

The online version of this article (doi:10.1007/s00259-015-3081-y) contains supplementary material, which is available to authorized users.

Granulomatosis with polyangiitis (GPA, Wegener’s) is an inflammatory disease entity affecting small to medium vessels. It is, together with microscopic polyangiitis (MPA) and eosinophilic granulomatosis with polyangiitis (EGPA, Churg-Strauss syndrome), characterized by the presence of antineutrophil cytoplasmic antibodies (ANCA) and they are frequently grouped together under the term ANCA-associated vasculitis (AAV) [1]. Sarcoidosis is a multisystemic disease with unknown aetiology [2]. These diseases share a common pathology of granuloma formation, although granulomas are noncaseating in sarcoidosis, while necrotizing in GPA.

Positron emission tomography (PET) scanning with 2-deoxy-2-[^18^F]fluoro-D-glucose (FDG) is used for detecting high glucose metabolism in malignancies and infectious and autoimmune diseases [3–5]. Recent studies have shown that PET scans show abnormalities in patients with ANCA-associated vasculitis [6, 7]. Coregistration with CT allows the increased FDG uptake to be localized to the underlying anatomy. Aortic FDG uptake had been described incidentally in patients undergoing PET scanning during oncology staging and its potential to measure arterial inflammation has since been investigated [8, 9]. The use of PET scanning has sparked attention in the field of atherosclerosis as the arterial FDG uptake may be an independent predictor of cardiovascular events. Moreover, PET scanning has been evaluated and proven to be an important diagnostic tool in large vessel vasculitis (LVV) [10–13]. Measuring arterial FDG uptake has proven to be a reliable and reproducible method when values are blood-normalized, also known as the target to background ratio (TBR) [14–16].

In our previous study we observed enhanced FDG uptake in the aorta of patients with GPA [17]. Pathologically enhanced FDG uptake may have implications for classification of the patients. At present, the distribution of vessel involvement dictates the differentiation between specifically defined forms of vasculitis [18]. Our previous study did not specifically address the aortic uptake of patients in AAV and aortic uptake was merely assessed in a qualitative manner [17]. The aim of our current study is to systematically assess aortic FDG uptake in patients with AAV compared to positive controls (LVV), disease controls (sarcoidosis) and healthy controls (HC) using ^18^F-FDG PET/CT.

## Materials and methods

### Patients

Retrospectively, 21 patients with GPA according to the 2012 revised International Chapel Hill Consensus Conference Nomenclature were included [19]. A PET scan was performed in patients with GPA during a period of suspected disease activity when other tools for evaluation of activity were inconclusive. The possibility of an active bacterial or viral infection was excluded by culture, serology and persistence of symptoms despite empirical antibiotic treatment. Previous renal involvement was determined by a renal biopsy showing pauci-immune necrotizing glomerulonephritis in the past [20]. However, the presence of haematuria in combination with red cell casts, dysmorphic erythrocytes (>10) and/or proteinuria sufficed [21].

Fourteen age- and sex-matched patients with biopsy-proven sarcoidosis according to the consensus statement on sarcoidosis of the American Thoracic Society (ATS)/European Respiratory Society (ERS)/World Association of Sarcoidosis and Other Granulomatous Disorders (WASOG) [2] were included as disease controls. The indication for a PET scan in patients with sarcoidosis was the presence of unexplained disease-related disabling symptoms that persisted for at least 1 year [4]. Seven age- and sex-matched patients in whom a PET scan was made during daily clinical practice for oncological staging of stage I or II head and neck carcinoma were included as HC. These patients had not (yet) received systemic chemotherapy or radiation therapy at the time of scanning. Lastly, five patients with giant cell arteritis (GCA) according to the 1990 criteria of the American College of Rheumatology (ACR) were included during a period of active disease as positive controls [22].

All PET scans were performed between December 2006 and March 2014 at the Maastricht University Medical Center. This study was carried out in compliance with the Helsinki Declaration; for this type of study formal consent is not required.

### ^18^F-FDG PET/CT

An ^18^F-FDG PET/CT scan was performed by scanning patients using a PET/CT (Gemini TF™ PET/CT, Philips Healthcare Nederland B.V., The Netherlands) scanner with time-of-flight (TOF) capability, together with a 64-slice Brilliance CT scanner. This scanner has a transverse and axial field of view (FOV) of 57.6 and 18 cm, respectively. The spatial resolution is around 5 mm. Patients were fasting for at least 6 h before the examination. In all patients blood glucose was measured to ensure that the blood glucose was below 10 mmol/l. FDG was injected intravenously and followed by physiological saline (10 ml). The injected total activity of FDG depended on the weight of the patient. The mean injected dose was 200 MBq. After a resting period of 45 min (time needed for uptake of FDG) PET and CT images were acquired from the head to the feet. A low-dose CT scan was performed without intravenous contrast and was used for attenuation correction of the PET images. The PET images were acquired in 5-min bed positions. The complete PET data set was reconstructed iteratively with a reconstruction increment of 5 mm to provide isotropic voxels.

Vascular PET image analysis was performed on a Xeleris 2.0 workstation (Extended Brilliance Workspace™ V4.5.3.40140, Philips Healthcare Nederland B.V., The Netherlands) using previously described, validated and reproducible methods [14, 15, 23, 24]. Briefly, the aorta was divided into ascending, arch, descending and abdominal segments using anatomical landmarks derived from the CT scan. The aorta was demarcated as the region of interest (ROI) on each slice of the CT scan. Maximum body weight-corrected standardized uptake values (SUV_max_) were derived from the pixel activity within the ROI on each slice of the PET scan. Slices were screened for nonvascular enhanced uptake in the close vicinity of the aorta that may falsely increase the SUV value, such as the heart or lymph nodes, and slices were censored if present. In addition, the mean blood FDG activity (SUV_mean_) was measured in the superior vena cava using an ROI of 5 mm on at least 5 slices, and the ROI was placed in the middle of the superior vena cava to ensure that no FDG uptake of the vessel wall was included. Arterial SUV_max_ values were blood-normalized by dividing the SUV_max_ of the aorta by the SUV_mean_ of the blood pool in the vena cava, known as the TBR_max_.

Aortic FDG uptake was evaluated using (1) the mean TBR_max_ of the whole aorta, (2) the mean TBR_max_ of the most diseased segment, defined as the hottest slice and the adjacent slices superior and inferior to it, and (3) the proportion of active slices, defined as a slice with a TBR higher than 1.6 [25–27].

### Statistical procedure

Numerical variables were expressed as mean (SD) or as median (interquartile range, IQR) and categorical variables as numbers (percentages). The Kruskal-Wallis test was used to compare quantitative measurements between more than two groups. Dunn’s multiple comparisons posttest analysis was performed to compare individual groups if a statistical difference was found in the Kruskal-Wallis test. To compare quantitative measurements between two unpaired groups, the Mann-Whitney test was performed. Continuous variables were correlated with the Spearman test.

To assess interrater reliability, a second reader performed the measurements and the results were compared using the intraclass correlation coefficient (two-way random and absolute agreement). All statistical analyses were performed using GraphPad Prism version 6.04 for Windows (GraphPad Software, La Jolla, CA, USA) and SPSS statistics for Windows, version 20.0 (IBM, Armonk, NY, USA).

## Results

Aortic FDG uptake using FDG PET/CT was assessed in 21 patients with GPA, 14 patients with sarcoidosis, 7 HC and 5 patients with LVV (see Table 1). In total, 89 (10), 97 (16), 97 (8) and 91 (5) slices of the aorta were evaluated in patients with GPA, sarcoidosis, HC and LVV, respectively, of which 2 (SD 4, 2.2 %), 8 (SD 8, 8.2 %), 4 (SD 5, 4.1 %) and 0 (SD 0, 0 %) slices were excluded due to adjacent nonvascular enhanced uptake. The interrater reliability with the second reader was excellent, with all intraclass correlation coefficient values higher than 0.9 (see Table 2).

**Table 1:** Patient characteristics at the time of scanning

	GPA	Sarcoidosis	HC	LVV
*n*	21	14	7	5
Age (years)	58.6 (16.8)	59.6 (10.3)	61.4 (13.2)	67.3 (7.3)
Female	14 (66.7 %)	6 (42.9 %)	2 (28.6 %)	5 (100 %)
BMI	24.4 (5.3)	27.8 (4.2)	24.1 (3.7)	24.7 (4.1)
Overweight	7 (33.3 %)	10 (71.4 %)	3 (42.9 %)	5 (100 %)
Serum glucose	5.7 (0.8)	5.7 (0.9)	5.7 (0.3)	5.5 (0.8)
Serum creatinine	84 (78–101)	78 (70–97)	77 (73–85)	73 (58–78)
C-reactive protein	6 (3–98)	4 (3–6)	3 (2–4)	44 (5–71)
Medication				
Hypertension	8 (38.1 %)	7 (50 %)	1 (14.3 %)	1 (20 %)
Diabetes	4 (19.0 %)	2 (14.3 %)	0 (0 %)	0 (0 %)
Statin	6 (28.6 %)	5 (35.7 %)	0 (0 %)	0 (0 %)
Prednisone	14 (66.7 %)	4 28.6 %)	0 (0 %)	1 (20 %)

Values are expressed as mean (SD), median (IQR) or *n* (%)

*BMI* body mass index

**Table 2:** Intraclass correlation coefficient values (95 % confidence interval)

	SUV_max_
Aorta abdominalis	1.00 (1.00–1.00)
Aorta descendens	0.99 (0.97–0.99)
Aortic arch	0.98 (0.96–0.99)
Aorta ascendens	0.97 (0.95–0.99)
Superior vena cava^a^	0.90 (0.68–0.96)

^a^The superior vena cava was measured as SUV_mean_

**Fig. 1:**
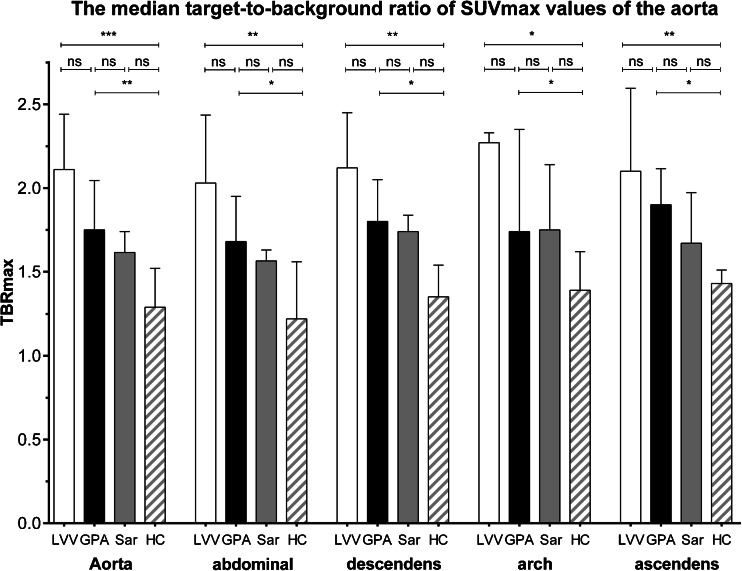
Median TBR of SUV_max_ values of the aorta and anatomical segments in patients with LVV, GPA, sarcoidosis (*Sar*) and HC. The *columns* and *brackets* represent the median and IQR. *p < 0.05; **p < 0.005; ***p < 0.0005

Differences were observed in the median mean TBR_max_ of the aorta between patients with LVV [2.03 (1.67–2.45)], patients with GPA [1.75 (1.32–2.05)], patients with sarcoidosis [1.62 (1.54–1.74)] and HC [1.29 (1.22–1.52)]. The mean TBR_max_ was higher in patients with LVV and GPA compared to HC (*p* < 0.005 and *p* < 0.05, respectively). The mean TBR_max_ tended to be higher in patients with sarcoidosis compared to HC, but this did not reach statistical significance (*p* = 0.098). This pattern was observed in all anatomical segments of the aorta (see Fig. 1). The median SUV_max_ values of the aorta were 3.47 (2.37–3.58) in patients with LVV, 1.90 (1.65–2.06) in patients with AAV, 2.03 (1.88–2.51) in patients with sarcoidosis and 1.62 (1.60–2.32) in HC. The median SUV_mean_ values of the superior vena cava were 1.38 (1.30–1.62), 1.09 (0.81–1.30), 1.25 (1.09–1.45) and 1.34 (1.10–2.09] in patients with LVV, AAV, sarcoidosis and HC, respectively.

**Fig. 2:**
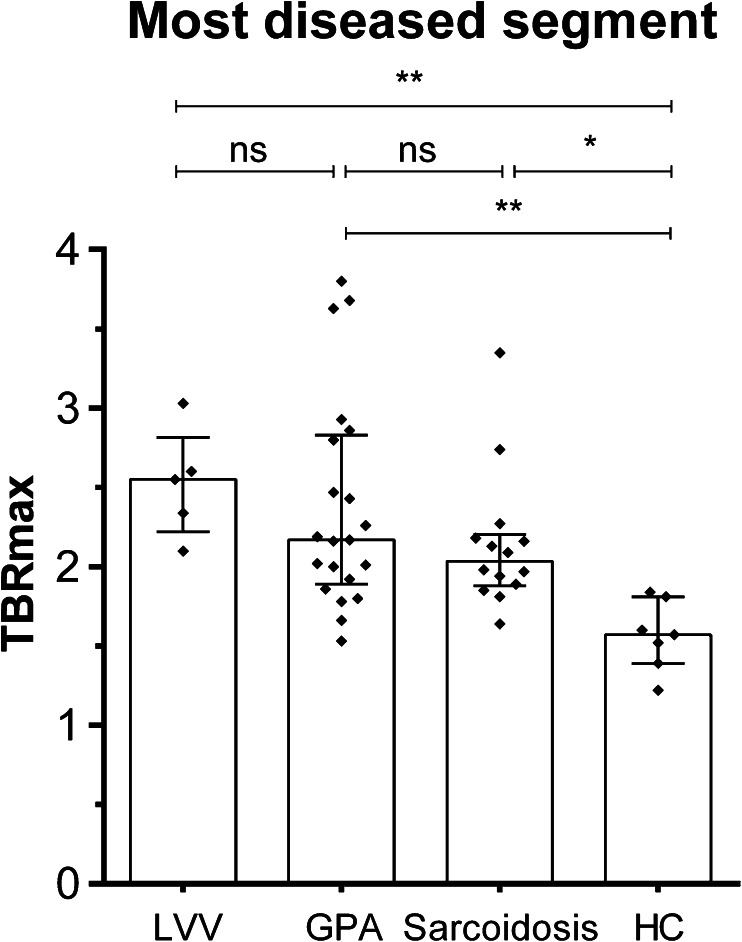
Median TBR of SUV_max_ values of the most diseased segment of the aorta in patients with LVV, GPA, sarcoidosis and HC. The *columns* and *brackets* represent the median and IQR. *p < 0.05; **p < 0.005

The median mean TBR_max_ of the most diseased segment was significantly higher compared to HC [1.57 (1.39–1.81)] in patients with LVV [2.55 (2.22–2.82), *p* < 0.005], patients with GPA [2.17 (1.89–2.83), *p* < 0.005] and patients with sarcoidosis [2.04 (1.88–2.20), *p* < 0.05, see Fig. 2].

**Fig. 3:**
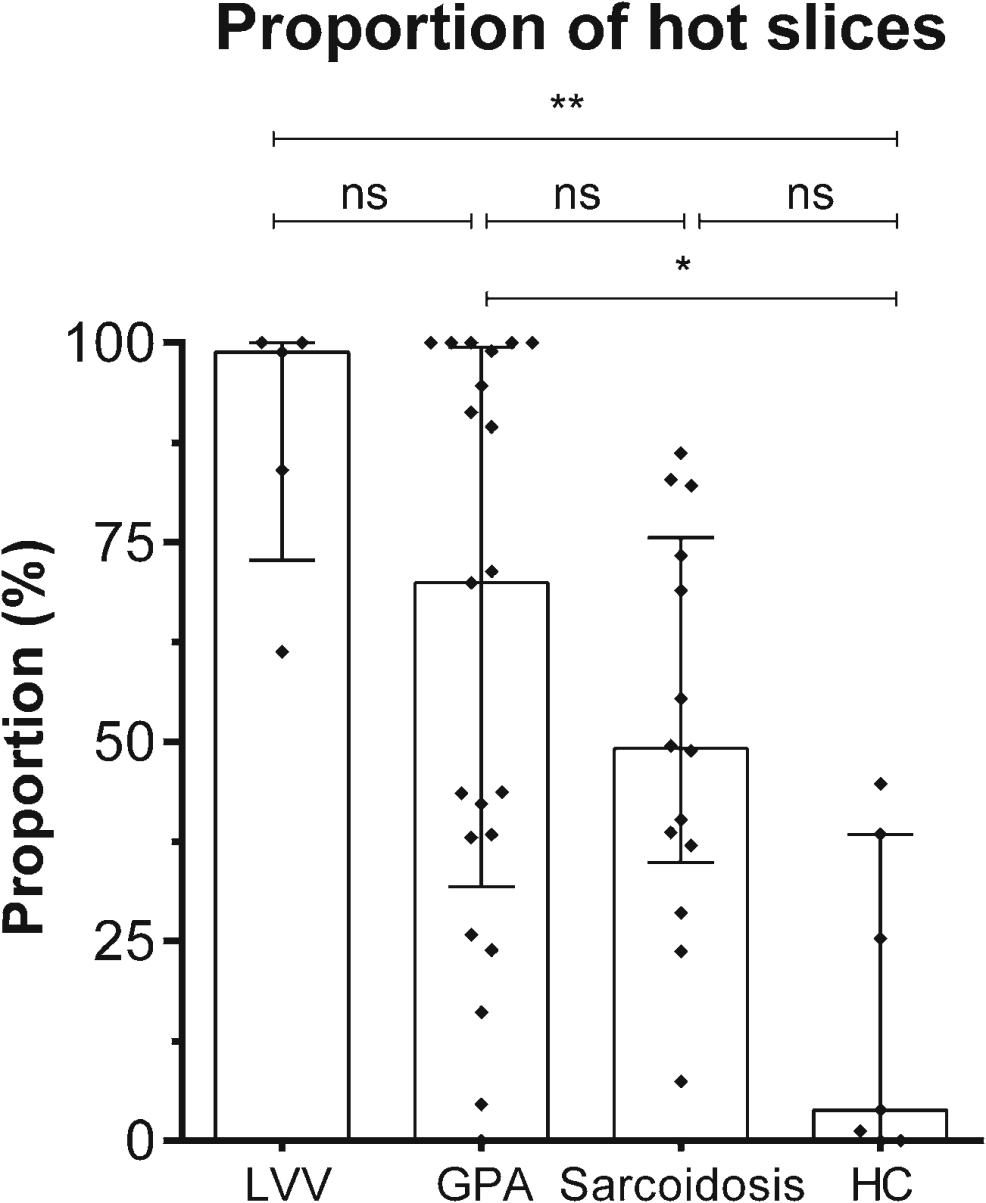
The proportion of hot slices (defined as TBR_max_ > 1.6) in patients with LVV, GPA, sarcoidosis and HC. The *columns* and *brackets* represent the median and IQR. *p < 0.05; **p < 0.005

The proportion of hot slices was significantly higher compared to HC [3.85 % (0–38.46)] in patients with LVV [98.85 % (72.69–100), *p* < 0.005] and patients with GPA [69.89 % (31.89–99.50), *p* < 0.05, see Fig. 3]. The proportion of hot slices was not statistically different in patients with sarcoidosis [49.17 % (34.89–75.58)] compared to HC (*p* = 0.21).

**Fig. 4:**
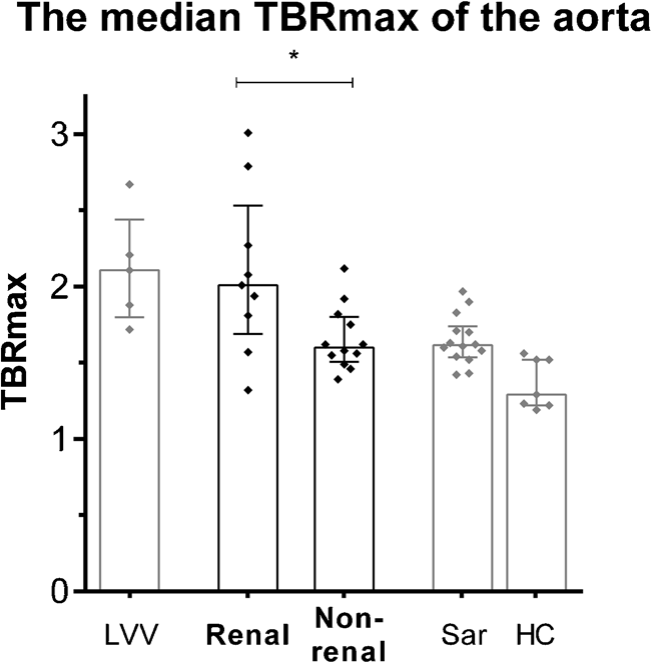
Difference in median TBR of SUV_max_ values of the aorta between GPA patients with renal involvement and GPA patients without renal involvement. *Sar* sarcoidosis. The *columns* and *brackets* represent the median and IQR. *p < 0.05

In patients with GPA, the median mean TBR_max_ of the aorta was significantly higher in patients with previous renal involvement [2.01 (1.69–2.53)] compared to patients without renal involvement in the past [1.60 (1.51–1.80), *p* < 0.05, see Fig. 4]. The mean TBR_max_ of the aorta correlated significantly with the C-reactive protein level at the time of scanning (*R* = 0.42, *p* < 0.005).

## Discussion

It was demonstrated that PET scans show higher FDG uptake in the aorta in patients with AAV compared to HC. In addition, the most diseased segment was higher in patients with GPA and patients with sarcoidosis compared to HC. Moreover, the aortic uptake in GPA patients with previous renal involvement was higher compared to patients without renal involvement in the past and similar to patients with LVV.

Since the accidental finding of aortic FDG uptake during oncological staging, ^18^F-FDG PET scanning has been advocated as an imaging tool that reflects the cellular pathology of atherosclerosis rather than the anatomical consequence [28]. The central question remains what underlying process is reflected by the enhanced FDG uptake in the aorta. Currently, the FDG uptake is thought to represent macrophage activity in inflamed intimal atherosclerotic plaques [9, 29]. Several histopathological studies have indeed shown that the intensity of FDG uptake correlates with the number of macrophages observed in the vessel wall [26, 28]. Interestingly, FDG uptake and arterial calcification rarely overlap [30].

The phenomenon of accelerated atherosclerosis is observed in various autoimmune diseases, including GPA [31]. Patients with GPA have a two- to fourfold increased relative risk of coronary artery disease as compared to control subjects, independent of classic risk factors [31]. Long-term mortality in GPA is dominated by cardiovascular events [32]. Recently we have shown that the metabolic syndrome is more prevalent in patients with AAV compared to HC [33]. More specifically, patients with AAV suffer more often from hypertension and an increased waist circumference, while the prevalence of the other criteria for the metabolic syndrome are comparable. In our current study, patients with sarcoidosis more often suffered from overweight and hypertension, while other cardiovascular risk factors were similar.

The finding of enhanced aortic FDG uptake has been studied in parallel in patients with LVV [34]. The spectrum of LVV consists of Takayasu’s arteritis (TA) and GCA. FDG PET scans have been performed in both diseases and have shown high SUV values of the aorta. However, most studies concerning LVV only report the SUV value, which may not be the optimal measure to quantify FDG uptake in the aorta [9, 24, 35]. The TBR_max_ value has the advantage, to a certain extent, to correct for the FDG uptake in the blood. Recently an effort has been made towards an optimal semi-quantitative approach to measure arterial inflammation in LVV [16]. Interestingly, SUV_max_ values of the aorta were not increased in patients with GPA compared to the other patient groups (see “Supplementary material”). This has been observed in patients with GCA as well, in which the TBR_max_ value, but not the SUV value, was significantly higher in patients with GCA compared to HC [16]. Normalization by blood pool activity outperformed the other approaches and permitted the differentiation of patients with GCA from control patients [16].

The distribution of vessel involvement has in the past dictated the differentiation between specifically defined forms of vasculitis [18]. Cases have previously been reported with evident large vessel involvement in patients with small vessel vasculitis, thereby questioning the validity of the differentiation in vessel distribution [36]. Therefore, in the 2012 revised Chapel Hill Consensus Conference, it is noted that there is substantial overlap with respect to arterial involvement [19]. Our results support this notion, as the mean TBR_max_ values in patients with AAV were comparable to the values measured in patients with GCA.

Does the enhanced aortic FDG uptake in our patients with GPA reflect atherosclerosis or large vessel involvement? The mean TBR_max_ values measured in our patients with GPA are at the high end of values observed in patients with established cardiovascular disease (see Table 3). Unfortunately, FDG uptake is not specific for processes of either disease. More importantly, should the finding of enhanced aortic FDG uptake prompt a change in treatment? Several studies have shown that the FDG uptake reduces after statin treatment [40, 38]. In addition, Mäki-Petäjä et al. have shown that anti-tumour necrosis factor-α treatment reduces aortic inflammation in patients with rheumatoid arthritis [25]. However, it is currently uncertain whether enhanced FDG uptake in the aorta should be an indication to start treatment with a statin or an immunosuppressive agent. Further research is warranted to assess the clinical significance of aortic inflammation.

**Table 3:** Mean TBR of the aorta in different subgroups of patients as reported in the literature

Study	Disease	Aorta	Abdominalis	Descendens	Arch	Ascendens
Rudd et al. (2007) [14]	CVD		1.30 (0.12)	1.23 (0.16)	1.31 (0.16)	1.39 (0.18)
Rudd et al. (2009) [37]	CVD		1.65	1.65	1.85	1.95
Coulson et al. (2010) [23]	COPD		2.14 (0.16)	2	2.1	2.2
	Ex-smoker		1.83 (0.20)	2	1.95	1.95
	MetS		2.5	2.5	2.5	2.5
Mäki-Petäjä et al. (2012) [25]	RA	2.02 (0.22)				
	CVD	1.74 (0.22)				
Wu et al. (2012) [38]	CVD	1.83 (0.37)				
	Control	1.49 (0.23)				
Noh et al. (2013) [39]	Control	1.60 (0.15)				
Besson et al. (2014) [16]	GCA			1.86 (0.45)	1.95 (0.43)	1.75 (0.37)
	Control			1.51 (0.30)	1.59 (0.33)	1.56 (0.33)
Kemna et al. (2015) [17]	GPA	1.84 (0.44)	1.79 (0.43)	1.86 (0.44)	1.93 (0.51)	2.00 (0.62)
	Sarcoidosis	1.65 (0.16)	1.58 (0.22)	1.68 (0.22)	1.92 (0.53)	1.73 (0.27)
	Control	1.36 (0.16)	1.34 (0.17)	1.38 (0.17)	1.40 (0.22)	1.41 (0.14)
	GCA	2.18 (0.36)	2.05 (0.41)	2.17 (0.33)	2.10 (0.32)	2.21 (0.44)

*CVD* cardiovascular disease, *COPD* chronic obstructive pulmonary disease, *MetS* metabolic syndrome, *RA* rheumatoid arthritis

Lastly, we have shown that the mean TBR_max_ value of the most diseased segment of the aorta in patients with sarcoidosis was higher compared to HC, while the mean TBR_max_ values of the entire aorta were comparable. This suggests that patients with sarcoidosis suffer from aortic inflammation with a focal pattern but not diffuse. Systemic vasculitis in sarcoidosis is uncommon but has been described previously [41].

Our study suffers from several limitations. Due to the retrospective character of the study we were unable to assess other risk factors, such as the lipid profile. However, in a previous study these values were not elevated in our patients with AAV compared to HC [33]. Our patients with stage I or II head and neck carcinoma suffered from less cardiovascular risk factors compared to the patients with GPA and may therefore not be an ideal control group. The mean TBR_max_ of 1.36 ± 0.16 observed in our HC was slightly lower compared to the values reported in the literature, ranging between 1.49 and 1.60. Recent findings suggest that the pre-scan glucose levels and the circulation times were suboptimal for the evaluation of FDG uptake in the aorta [24].

In conclusion, patients with GPA showed marked FDG uptake in the aorta compared to HC. In addition, the aortic FDG uptake in GPA patients with previous renal involvement was equivalent to the aortic FDG uptake in patients with LVV.

## Electronic supplementary material

ESM 1 (DOCX 698 kb)
