# Supplementary material for: Aortic 18F-FDG uptake in patients suffering from granulomatosis with polyangiitis
Source: Eur J Nucl Med Mol Imaging. 2015 May 21;42(9):1423–9. doi: 10.1007/s00259-015-3081-y (PMC4502318; doi:10.1007/s00259-015-3081-y)
Supplement: Supplementary file 1 — (DOCX 698 kb) [file 259_2015_3081_MOESM1_ESM.docx]

**Aortic ^18^F-FDG uptake in patients suffering from Granulomatosis with Polyangiitis – Supplemental materials**

**Running title: Aortic inflammation in GPA**

**Michael J. Kemna^a,b^, Jan Bucerius^b,c,d^, Marjolein Drent^e^, Stefan Vöö^b,c^, Martine Veenman^b^, Pieter van Paassen^a^, Jan Willem Cohen Tervaert^b,f^, Marinus J.P.G. van Kroonenburgh^c^**

*^a^Department of Nephrology and Clinical Immunology, Maastricht University Medical Center, Maastricht, The Netherlands*

*^b^Cardiovascular Research Institute Maastricht (CARIM), Maastricht University, Maastricht, The Netherlands*

*^c^Department of Nuclear Medicine, Maastricht University Medical Center, Maastricht, The Netherlands*

*^d^Department of Nuclear Medicine, University Hospital RWTH Aachen, Aachen, Germany*

***^e^****Department of Pharmacology and Toxicology, Maastricht University, Maastricht, The Netherlands*

*^f^Noordoever Academy, Sint Franciscus Gasthuis, Rotterdam, The Netherlands*

**Results:**

**SUVmax values**

The median SUVmax values of the aorta are 3.47 [2.37-3.58] in patients with LVV, 1.90 [1.65-2.06] in patients with AAV, 2.03 [1.88-2.51] in patients with sarcoidosis and 1.62 [1.60-2.32] in “healthy” controls. The median SUVmean values of the vena cava superior are 1.38 [1.30-1.62], 1.09 [0.81-1.30], 1.25 [1.09-1.45] and 1.34 [1.10-2.09] in patients with LVV, AAV, sarcoidosis and “healthy” controls, respectively.

**

**

**Figure S1.** Median SUVmax values of the aorta and anatomical segments in patients with LVV, GPA, sarcoidosis (Sarc) and healthy controls (HC). The column and brackets represent the median and interquartile range. * p < 0.05, ** p < 0.005.
